# Micronutrient Fortified Milk Improves Iron Status, Anemia and Growth among Children 1–4 Years: A Double Masked, Randomized, Controlled Trial

**DOI:** 10.1371/journal.pone.0012167

**Published:** 2010-08-13

**Authors:** Sunil Sazawal, Usha Dhingra, Pratibha Dhingra, Girish Hiremath, Archana Sarkar, Arup Dutta, Venugopal P. Menon, Robert E. Black

**Affiliations:** 1 Johns Hopkins Bloomberg School of Public Health, Johns Hopkins University, Baltimore, Maryland, United States of America; 2 Department of Biochemistry, Center for Micronutrient Research, Annamalai University, Chidambaram, India; Institute of Clinical Effectiveness and Health Policy, Argentina

## Abstract

**Background:**

Multiple micronutrient deficiencies are highly prevalent among preschool children and often lead to anemia and growth faltering. Given the limited success of supplementation and health education programs, fortification of foods could be a viable and sustainable option. We report results from a community based double-masked, randomized trial among children 1–4 years evaluating the effects of micronutrients (especially of zinc and iron) delivered through fortified milk on growth, anemia and iron status markers as part of a four group study design, running two studies simultaneously.

**Methods and Findings:**

Enrolled children (n = 633) were randomly allocated to receive either micronutrients fortified milk (MN = 316) or control milk (Co = 317). Intervention of MN milk provided additional 7.8 mg zinc, 9.6 mg iron, 4.2 µg selenium, 0.27 mg copper, 156 µg vitamin A, 40.2 mg vitamin C, and 7.5 mg vitamin E per day (three serves) for one year. Anthropometry was recorded at baseline, mid- and end-study. Hematological parameters were estimated at baseline and end-study. Both groups were comparable at baseline. Compliance was over 85% and did not vary between groups. Compared to children consuming Co milk, children consuming MN milk showed significant improvement in weight gain (difference of mean: 0.21 kg/year; 95% confidence interval [CI] 0.12 to 0.31, p<0.001) and height gain (difference of mean: 0.51 cm/year; 95% CI 0.27 to 0.75, p<0.001). Mean hemoglobin (Hb) (difference of 13.6 g/L; 95% CI 11.1 to 16.0, p<0.001) and serum ferritin levels (difference of 7.9 µg/L; 95% CI 5.4 to 10.5, p<0.001) also improved. Children in MN group had 88% (odds ratio = 0.12, 95% CI 0.08 to 0.20, p<0.001) lower risk of iron deficiency anemia.

**Conclusions/Significance:**

Milk provides an acceptable and effective vehicle for delivery of specific micronutrients, especially zinc and iron. Micronutrient bundle improved growth and iron status and reduced anemia in children 1–4 years old.

**Trial Registration:**

ClinicalTrials.gov NCT00255385

## Introduction

Undernutrition and anemia impact health, growth, development and survival of young children in the low-income countries [1], [2]. Growth stunting affects 182 million children younger than five years old and over 50% of children have anemia [3], [4]. Deficiency of essential micronutrients is one of the commonest attributable causes [5], [6]. Micronutrient deficiency in turn can increase the risk and/or severity of morbidities and anorexia, thereby further depleting micronutrient stores, adversely affecting the hematological parameters and impairing physical growth [4], [7], [8]. In first three years of life, hematopoesis and rapid growth represent a highly active metabolic state requiring several micronutrients thus making them vulnerable to deficiency. Recent evidence suggests that delivery of specific multiple micronutrient combinations as compared to single micronutrient may have a greater beneficial effect on iron stores, hematological parameters and growth [9]–[12].

National level supplementation programs and health education to change dietary practices in preschool children have achieved limited success in alleviating micronutrient deficiencies in vulnerable populations. Implementation costs, frequency of administration and hence lack of adherence to supplementation, complexities of changing feeding behavior and lack of options that would deliver optimal bio-available iron and zinc [13]–[15] could be the reasons for limited success of supplementation programs. On the other hand, encouraging results from fortification intervention trials and the national level fortification programs (in Brazil and Chile) have identified fortification of common home-based foods as an attractive alternative [16]–[18].

We undertook a community-based, doubled-masked randomized trial with four arms to evaluate the effect of two different milk interventions in comparison to their respective control groups (essentially running two trials concurrently with a common randomization). Two groups evaluated impact of fortifying regular milk powder with micronutrient bundle in comparison to same milk without fortification; and the other two groups evaluated fortification of pre-fortified premium milk with prebiotic and probiotic in comparison to same milk without pre and probiotics fortification. In this paper we are reporting the results of the two arms evaluating efficacy of consumption of multiple micronutrients (including iron and zinc) fortified milk for a period of one year on growth, body iron stores and anemia among children aged 1–4 years. The results of the other two arms are reported separately in a companion paper [19].

## Methods

The protocol for this trial and supporting CONSORT checklist are available as supporting information; see Checklist S1 and Protocol S1.

### Participants

The trial was carried out from April 2002 to April 2004, in Sangam Vihar, a peri-urban community located on the outskirts of New Delhi, India. Detailed population description has previously been reported [20]. Center for Micronutrient Research has been maintaining a regularly updated comprehensive demographic database of Sangam Vihar for over a decade. From the database, all permanent resident families in the area with children aged 1–3 years were invited to participate in the study, and their consent sought. Children who were exclusively or predominantly breast fed or allergic to milk were excluded. Children with severe malnutrition needing rehabilitation or chronic/severe illness requiring hospitalization or special treatment were to be excluded; however, none of the children contacted met this criterion. At enrollment, all children who had severe anemia (hemoglobin [Hb] <70 g/L) were given therapeutic dose of iron for 3 months in addition to their assigned intervention.

### Ethics

The human research and ethical review committees at the Johns Hopkins University, USA and the Annamalai University, India approved the study protocol. The purpose of the study along with the possible risks and benefits associated was read out and explained to the parents in the local language, and informed written consent obtained. Procedure consisted of supervisor visiting the household and in presence of a third party, obtaining the consent from the mother or father after reading the consent form to them. Parents were given a choice to sign the consent form or if they were illiterate and/or could not sign, supervisor and the witness signed to document the consent.

This procedure had been approved by both institutional review boards' as majority of the mothers cannot sign in this population and taking thumb impression is stigmatized due to misuse during colonial era.

### Procedures

Mothers were requested to bring the child to the study clinic for baseline assessment. At enrolment, the study physician carried out a detailed physical examination of the child and collected the socio-economic and demographic information of the household.

Weight was measured to the nearest 10 g with an electronic scale (SECA Corporation, Columbia, MD/ATCO weighing Solutions Company Limited, India) by two independent observers, and height was recorded to nearest 0.1 cm using height boards (Shorr Productions, Olney, MD). The weight and height of the child was measured at enrollment, after 6 months (mid-study) and one year of intervention (end-study).

A venous blood sample was collected using a trace element-free syringe, immediately transferred into ethylenediaminetetraacetic acid vial for a detailed hemogram and zinc protoporphyrin (ZnPP) analysis, and zinc-free heparin vials for plasma zinc estimation. Plasma was separated within 15 minutes of blood collection, and aliquots were transferred into trace element-free Eppendorf plastic tubes for storage at −20°C. The ethylenediaminetetraacetic blood was analyzed on the same day with a Coulter automated flow cytometer (Beckman-Coulter, Fullerton, CA) for a detailed hemogram. One drop of blood was used for estimating ZnPP using a front face hematofluorometer (Aviv Biomedical, Lakewood NJ). Plasma zinc estimations were performed by standard methods using atomic absorption spectrometer (AAS 800-Perkin Elmer) [21]. Serum ferritin (sFr) and serum transferrin (sTfr) were estimated from plasma samples using a commercial enzyme linked immunosorbent assay kit (Spectro Ferritin Kit, Spectro Transferrin Kit; Ramco Laboratories, Inc., Houston, Texas). Biochemical analyses were repeated after one year of intervention.

### Intervention

Fonterra Brands (Singapore) Pte. Ltd. provided 32 g single serve sachets of fortified milk powder and control for the study. At enrollment, the procedure for preparing milk was clearly explained and demonstrated to mothers. Each week, the milk assistants delivered 21 sachets at home and advised the mother to feed the child 3 sachets a day. Composition of milk in intervention and control groups is given in Table 1. Fortified milk (3 servings per day) was designed to deliver additional amounts of zinc (7.8 mg), iron (9.6 mg), selenium (4.2 µg), copper (0.27 mg), vitamin A (156 µg), vitamin C (40.2 mg), vitamin E (7.5 mg) while the control milk powder provided natural levels of the specific micronutrients as in base milk without additional fortification of zinc or iron. Intervention continued for a year. Mothers were advised to continue breast-feeding for children who were breastfed and to continue giving whatever other supplementary feeding the child was taking. The timing of milk feeds in relation to other feeding was not controlled and was decided by mother.

**Table 1:** Composition of micronutrient fortified milk vs. control milk (cheap regular milk used as base milk).

Nutritive value (3 serves per day)	Micronutrient Milk Group (MN)	Control Group (Co)
Energy (kJ)	1890	1890
Protein (g)	20.1	20.1
Taurine (mg)	48	48
Carbohydrate (g)	48.9	48.9
Fat (g)	18.9	18.9
**Vitamin A** a (µg)	**330**	**174**
Vitamin D_3_ (µg)	3.6	3.6
**Vitamin E** b (mg)	**8.1**	**0.6**
**Vitamin C** (mg)	**48**	**7.8**
Thiamin (mg)	0.6	0.6
Riboflavin (mg)	1.8	1.8
Niacin (mg)	4.5	4.5
Vitamin B_6_ (mg)	0.6	0.6
Pantothenic Acid (mg)	2.7	2.7
Folate (DFE)^c^ (µg)	114	114
Vitamin B_12_ (µg)	2.7	2.7
Biotin (µg)	24.9	24.9
Choline (mg)	114	114
Calcium (mg)	720	720
Phosphorus (mg)	600	600
Magnesium (mg)	84	84
**Iron** (mg)	**9.6**	**0**
**Zinc** (mg)	**9.6**	**1.8**
Iodine (µg)	36	36
**Selenium** (µg)	**6.6**	**2.4**
**Copper** (mg)	**0.3**	**0.03**
Sodium (mg)	360	360
Potassium (mg)	1260	1260
Chloride (mg)	900	900

a Retinol activity equivalents.

b α - tocopherol equivalents.

c Dietary folate equivalents.

**Milk Ingredients:** Skim milk, added carbohydrate, Cream, Sucrose, Lactose, Corn oil, Lecithin, Vanillin, **Vitamins:** Vitamin A, Vitamin D_3_, Vitamin E, Thiamin hydrochloride, Pyridoxine hydrochloride, Vitamin C, Folate, Niacinamide, **Minerals:** Ferrous sulphate, Zinc sulphate, Copper sulphate (Fortified milk contains additional fortification of Zinc, Iron, Vitamin A, Vitamin E, Vitamin C, Selenium and Copper).

### Outcomes

Primary outcomes were not explicitly pre-specified in the protocol; the intent was to evaluate the impact on morbidity, growth, development, iron status and anemia. However, the sample size was estimated based on the effects on diarrhoea and pneumonia. We have already reported the effect of micronutrient-fortified milk on the morbidity outcomes [20]. In this paper we are reporting the effect on growth, anaemia and iron status.

The effect on growth parameters was evaluated by estimating the mean change in Z-scores at end-study from baseline and gain in weight/height over a period of 1 year of supplementation. Stunting was defined as height for age (HAZ) <−2 Z-scores, wasting as weight for height (WHZ) <−2 Z-scores and underweight as weight for age (WAZ) <−2 Z-scores [22]. The effect of intervention compared to control on iron status and anemia was evaluated by estimating the difference of means from baseline to end-study for each of the hematological parameters [Hb, Hematocrit (Hct), ZnPP, Red cell distribution width (RDW), sFr, sTfr] and the change in proportion of iron deficient anemic children from baseline to end-study.

Children with Hb ≤100 g/L were considered anemic, and iron deficient state was defined as the presence of 2 or more of the following criteria: sFr ≤12µg/L, sTfr >8.3 µg/ml, Hct ≤30% and ZnPP ≥80 µmol per mole of heme. Iron deficiency anemia was categorized as Hb ≤100 g/L and the presence of two or more of four conditions of iron deficiency.

### Sample Size and Power

The sample size estimation for morbidity related outcomes, which was highest and drove sample size, has been described previously [20]. The sample size was estimated based on the assumption that micronutrient bundle would decrease diarrhea incidence by 15% and episodes of pneumonia by 25% with alpha of 0.05 and 90% power. This sample size provided a power of more than 90% to detect a change in anthropometric indices of 0.5 Z-score over the baseline Z-scores, at α = 0.05. It also provided a power of more than 90% to detect a change in mean Hb level by 5.0 g/L over the baseline Hb.

### Randomization: Sequence Allocation and Allocation Concealment

The study consisted of two trials run concurrently with common randomization. Letter codes A through D were used to identify four groups (across two separate trials). In-house computer software generated a random sequence of group codes with permuted block length of 16. Two separate randomization lists - one for children with baseline Hb >70 g/L and another for children with baseline Hb ≤70 g/L were used. After enrollment, each child was classified into two strata based on their baseline Hb and allocated the next available serial number and the letter code within that hemoglobin strata.

### Blinding and Implementation

The supplementation sachets were identical in colour, size (weight 32 g), taste and packaging and were labelled with a letter code. The investigators and the study team were blinded to the identity of the letter codes. Fonterra Brands Pte. Ltd. provided code identification to investigators after finishing of the trial, at the time of analysis. In the field, the letter code of the supplementation sachets was covered with label providing child's identification information.

### Data Management and Statistical Analysis

We used Visual Basic 6.0 and Oracle 8i to manage our data, with stringent range, consistency, and logical checks. Real time data entry ensured data quality and accuracy. We used double data entry and manual checking of frequencies during data cleaning before the code was broken. For primary analysis, we used alphabetical codes for groups still blinded to real group identity. The groups were assessed for comparability at baseline. The statistical significance for the group differences of continuous variables was established using a Student's t-test, and of categorical variables by chi-square test. We applied logistic regression using WAZ <−2.0 Z-score ‘Yes/No’ as dependent variable and baseline WAZ scores and group allocation as independent variables. Stratified analysis based on gender and age was carried out to understand the gender and age differences between the intervention and control groups. Multiple linear regression models were designed to examine the independent effect of fortification on weight and height gain after adjusting for other covariates like SES, maternal education, gender, age at baseline, baseline plasma zinc levels and anemia status.

The analyses were performed in STATA 9.2 (Stata Corp., College Station, Texas, USA), and SPSS 12.0 (SPSS Inc., Chicago, Illinois, USA). Anthropometric Z-scores were calculated using WHO standards [22]. Intent to treat analysis was performed and all children enrolled were included in the analysis, irrespective of compliance. For withdrawals, we included all data available until withdrawal.

## Results

In this paper we are presenting the results of one year intervention of micronutrient fortified milk on growth and iron indices of children allocated to two of the four letter codes i.e. micronutrient fortified milk (MN) and its control (Co).

### Participants

**Figure 1:**
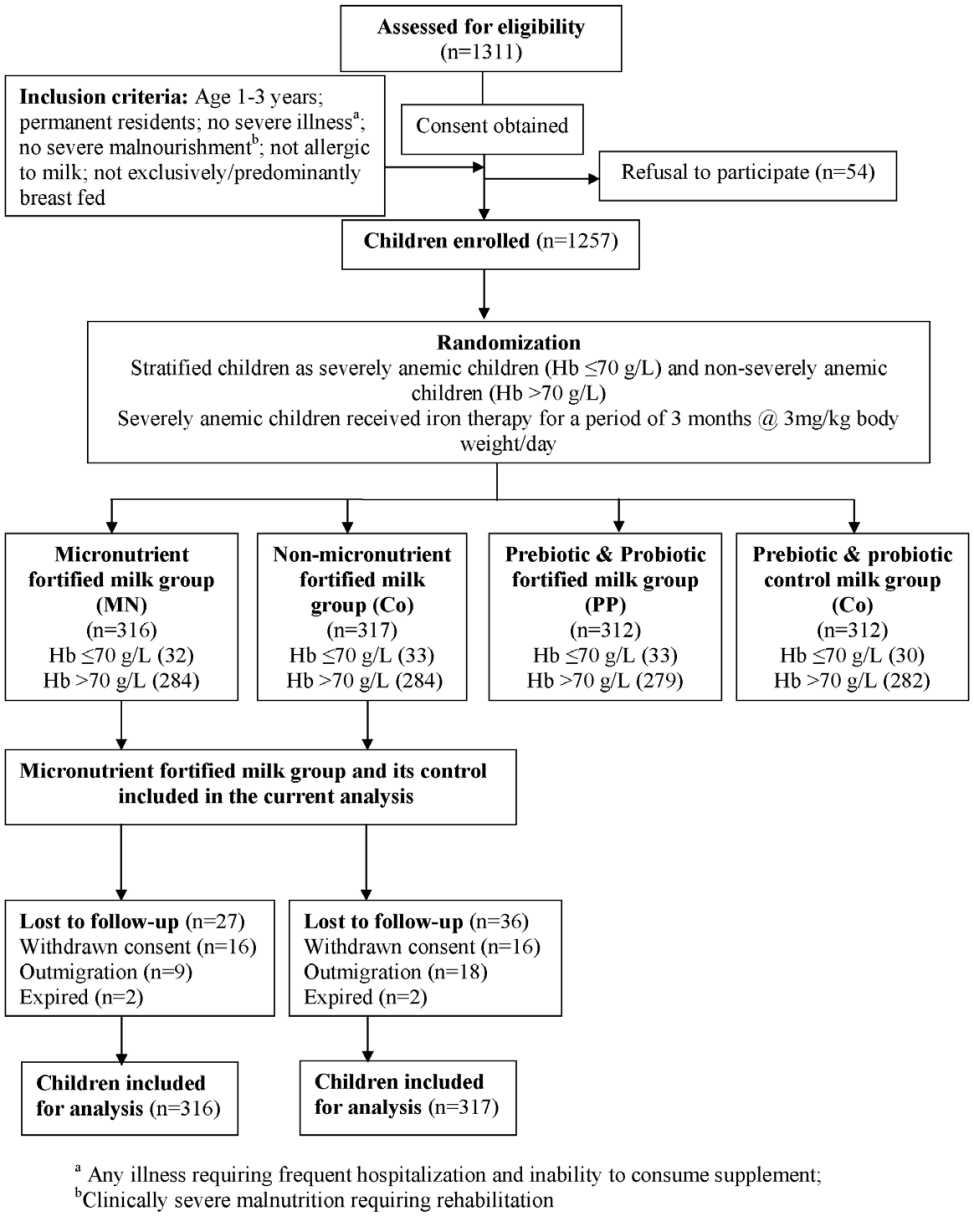
Schematic flow of the participants in the study.

Out of 660 eligible children, 27 (parents/caretakers) refused to participate. The enrolled children were randomly allocated to receive either micronutrient-fortified milk (MN group; n = 316) or the same milk with natural levels of micronutrients (Co group; n = 317) (Figure 1).

### Baseline Data

At baseline, the study groups were comparable for socio-economic, demographic, hematological and anthropometric parameters (Table 2). Among enrolled children, 66.8% in MN and 62.4% in Co group were moderately anemic (Hb: 70.1–100 g/L); 87.4% in MN and 85.7% in Co group had signs of iron deficient hematopoesis (ZnPP: >80 µmol/mole of heme); and 58.2% in MN and 50.3% in Co group were iron deficient anemic. Almost 68% of children were experiencing growth faltering.

**Table 2:** Baseline characteristics of children included in the study.

Variables	Category	MN Group (n = 316)	Co Group (n = 317)
**Age** (mo)^a^	-	22.4±6.8	23.0±6.7
**Illiterate father** b	-	54 (17.1)	52 (16.4)
**Illiterate mother** b	-	168 (53.2)	169 (53.3)
**Occupation father**	Daily wage labor^b^	114 (36.1)	106 (33.4)
**Occupation mother**	Housewives^b^	308 (97.5)	307 (96.8)
**Socio-economic status score** a	-	7.1±2.7	7.3±2.5
**Hematological markers** c	Hemoglobin^a^ (g/L)	89.2±15.0	91.0±14.9
	Hematocrit^a^ (%)	30.3±4.0	31.0±4.4
	Zinc protoporphyrin^a^ (µmol/mole of heme)	214.4±134.5	212.7±135.3
	Red cell distribution width^a^ (%)	19.4±2. 8	19.2±2.6
	Sr ferritin^a^ (µg/L)	8.6±8.0	9.3±8.2
	Sr transferrin^a^ (µg/ml)	15.6±9.1	14.9±9.1
	Iron deficient anemic^b^^,^ d	171 (58.2)	154 (50.3)
**Plasma zinc** a (µg/dL)	-	60.7±23.3	62.6±26.8
**Anthropometric parameters**	Weight^a^ (kg)	9.08±1.5	9.03±1.5
	Height^a^ (cm)	78.2±5.7	78.0±5.8
	Normal^b^	102 (32.3)	104 (32.8)
	Wasted and stunted^b^	55 (17.4)	46 (14.6)
	Stunted^b^^,^ e	149 (47.0)	147 (46.5)
	Wasted^b^^,^ e	10 (3.2)	20 (6.3)

a Mean±standard deviation.

b Number (%).

c The cut offs for defining anemia and iron deficiency that are conventionally used are Hb ≤100 g/L, Hct ≤30%, ZnPP ≥80 µmol per mole of heme, RDW >14%, SFr ≤12µg/L, STfr >8.3 µg/ml.

d At least 2 parameters for defining iron deficiency were available in only 294 children in MN group and 306 children in Co group.

e Stunting refers to stunting only, ie without wasting and wasting refers to wasting only, ie without stunting.

### Compliance

The compliance (adherence to study milk feeds) was assessed by collecting the information on consumption of milk and the previous week's remaining supply of milk sachets during the one-year intervention period. The median adherence to the intervention was over 85% in both groups. The mean number of sachets consumed per day was 2.58 in the intervention group and 2.54 in the control group. No adverse episode due to consumption of milk was reported during the course of the study.

### Effect on Growth

Compared to children consuming the non-fortified milk, children consuming MN milk, on an average gained 0.21 kg/yr (95% CI 0.12 to 0.31, p<0.001) in weight and 0.51 cm/yr (95% CI 0.27 to 0.75, p<0.001) in height. After 1 year of intervention, children in the fortified milk group showed a significant improvement in the WHZ, WAZ and HAZ scores (Table 3). Corrected for baseline Z-score, consumption of fortified milk resulted in a significant decrease in the proportion of children with WAZ <−2 Z-scores [MN vs. Co: odds ratio = 0.63 (95% CI 0.40 to 0.99), p = 0.05]. The growth effects observed at 6 months are presented in supplementary Table S1.

**Table 3:** Effect of fortification on anthropometric parameters among fortified milk vs. control milk group children after 1 year of intervention.

Variables	MN Group (n = 267)^a^	Co Group (n = 257)^a^	Difference of means (95% CI)	p value
**Weight velocity** c (kg/yr)	2.13±0.58^b^	1.92±0.55	0.21 (0.12 to 0.31)	<0.001
**Height velocity** d (cm/yr)	8.60±1.40	8.10±1.37	0.51 (0.27 to 0.75)	<0.001
**Change in Z-scores between baseline and end-study**				
Difference in WHZ score^e^	0.42±0.65	0.30±0.65	0.13 (0.02 to 0.24)	0.03
Difference in WAZ score^f^	0.38±0.54	0.18±0.51	0.20 (0.11 to 0.29)	<0.001
Difference in HAZ score^g^	0.28±0.47	0.09±0.42	0.19 (0.12 to 0.27)	<0.001

a End-study anthropometry data were available for 267 and 257 children in the MN and Co group respectively.

b Mean±SD.

c Rate of gain in body weight over a period of 1 year of intervention.

d Rate of gain in height over a period of 1 year of intervention.

e Weight for height Z-score.

f Weight for age Z-score.

g Height for age Z-score.

After adjusting for the socio-economic status, maternal education, gender, baseline age, anemia and zinc status, children in the fortified group on an average gained more weight (0.23 kg/yr; 95% CI 0.12 to 0.33, p<0.001) and height (0.58 cm/yr; 95% CI 0.33 to 0.82, p<0.001) (Table 4).

**Table 4:** Multiple linear regression^a^ evaluating the effect of micronutrient fortification on weight velocity and height velocity of children after 1 year of intervention.

Variables	Weight velocity (n = 425)			Height velocity (n = 425)		
	B	95% CI	p value	B	95% CI	p value
Micronutrient fortification	0.23	0.12 to 0.33	<0.001	0.58	0.33 to 0.82	<0.001
Socio-economic status	−0.01	−0.03 to 0.008	0.23	0.04	−0.003 to 0.09	0.07
Maternal education	−0.18	−0.18 to 0.03	0.15	0.02	−0.23 to 0.27	0.87
Gender	−0.006	0.00 to 0.09	0.91	−0.11	−0.36 to 0.14	0.38
Age at baseline	−0.02	−0.03 to −0.01	<0.001	−0.08	−0.096 to −0.06	<0.001
Plasma zinc at baseline	−0.004	−0.006 to −0.002	0.001	−0.003	−0.008 to 0.002	0.23
Severe anemia	0.16	−0.03 to 0.34	0.09	0.36	−0.07 to 0.78	0.10
R^2^ (model summary)	0.11	-	<0.001	0.18	-	<0.001

a Adjusted for socio-economic status, maternal education, gender, age at baseline, plasma zinc levels at baseline and anemia status at baseline.

### Effect on Body Iron Stores and Anemia

Compared to children consuming control milk, children consuming fortified milk had significant improvement in anemia and repletion of body iron stores (Table 5). Mean Hb levels increased by 13.6 g/L (95% CI 11.1 to 16.0, p<0.001) in the fortified milk group as compared to the control group. Consumption of fortified milk resulted in 88% decrease in the proportion of children with iron deficiency anemia (95% CI 80% to 92%, p<0.001).

**Table 5:** Effect of micronutrient fortified milk on hematological parameters and plasma zinc status among fortified milk vs. control milk group children after 1 year of intervention.

Variables	MN Group (n = 233)^a^	Co Group (n = 232)^a^	Difference of means (95% CI)	p value
Hb^c^ (g/L)	109.0±11.4^b^	95.4±15.4	13.6 (11.1 to 16.0)	<0.001
Hct^d^ (%)	35.9±3.2	32.6±4.0	3.3 (2.7 to 4.0)	<0.001
ZnPP^e^ (µmol/mole heme)	79.7±68.8	176.8±130.9	−97.1 (−116.1 to −78.0)	<0.001
RDW^f^ (%)	16.0±2.1	18.5±2.6	−2.5 (−2.9 to −2.1)	<0.001
sFr (µg/L)	15.3±11.2	9.8±8.6	5.5 (3.5 to 7.4)	<0.001
sTfr (µg/ml)	7.3±4.4	11.7±7.0	−4.4 (−5.5 to −3.3)	<0.001
Plasma zinc (µg/dL)	61.4±26.8	63.4±29.4	−1.97 (−7.3 to 3.3)	0.47
Iron deficient anemic	31 (13.3)^g^	128 (55.2)	0.12 (0.08 to 0.20)^h^	<0.001

a End-study hematological data and plasma zinc levels were available for 233 and 232 children in the MN and Co group respectively.

b Mean±standard deviation.

c Hemoglobin.

d Hematocrit.

e Zinc protoporphyrin.

f Red cell distribution width.

g Number (%).

h Odds ratio (95% CI).

The improvement in Hb levels was consistent with improvement in hematocrit and erythrocytic markers. Fortified milk resulted in significant lowering of mean ZnPP, mean RDW and an increase in mean hematocrit (Table 5). Fortified milk supplementation resulted in a decrease in proportion of children with markedly elevated ZnPP levels (>80 µmol/mole of heme; MN vs. Co: 33.5% vs. 72%) and elevated RDW levels (RDW >14%; MN vs. Co: 85.4% vs. 97%). Fortified milk consumption compared to consumption of control milk, resulted in an increase in mean body iron stores (sFr) and a significant reduction in mean total iron binding capacity (sTfr) (Table 5).

The overall changes in the serological markers, erythrocytic indices, and the hemoglobin resulted in a significant reduction in the proportion of iron deficient children [MN vs. Co: 68 (29.2%) vs. 164 (70.7%), p<0.001].

Subgroup analyses by age and gender did not reveal any differential effect (data not presented) of fortified milk on hematological parameters.

## Discussion

Our results suggest that milk is a good and well-accepted vehicle for the delivery of zinc, iron and other micronutrients in young children. Similar to other fortification trials, we also observed high compliance rate over 1 year study period. The effect of milk fortified with specific micronutrients (zinc, iron, selenium, copper, vitamins A, C and E) compared to control milk for 1 year on growth, Hb levels, body iron stores in this study is a conservative estimate as both groups got 3 servings of milk in addition to their regular diet. In the interpretation of results we need to consider, that the base milk used was regular cheap milk with no added iron and only 1.8 mg of zinc (Table 1).

There is inconclusive evidence about the impact of iron and zinc fortification on anemia and iron status. Meta-analysis of 21 data sets from iron supplementation randomized controlled trials in children aged 0 to 12 years reported a significant difference in the mean change in Hb concentrations between treatment and control groups of 7.8 g/L, or an effect size of 1.49 (95% CI 0.46 to 2.51) [23]. In our trial, we observed a larger impact on iron status markers with iron fortification than in iron supplementation trials. Among children consuming fortified milk, the median Hb levels improved by 17 g/L compared to their baseline Hb levels. Similar beneficial effects have been reported by other fortification trials conducted in low-income countries [12], [18], [24]–[26]. Availability of vitamin A and other co-factors like riboflavin, B-12 and folic acid essential for erythropoiesis [27] could have resulted in larger overall improvement of hematological parameters. Presence of vitamin C in the fortified micronutrient bundle, potentiating bioavailability of iron may have contributed to this impact.

Although available data from published trials are not conclusive, it is postulated that zinc may reduce the beneficial effects of iron on iron status [11]. The observed effect on iron status markers suggests that delivering zinc and other micronutrients with iron via fortified food either enhances (in the presence of ascorbic acid) or does not interfere with the absorption and bioavailability of iron. In our trial, we did not observe any difference in plasma zinc concentrations between the groups. Whether that is due to negative impact of iron on bioavailability of zinc or limitation of plasma zinc not being a good marker of zinc status can only be surmised. The beneficial effects on morbidity reported earlier [20], observed anthropometric outcomes, which are commonly attributable to zinc intake rather than iron [9], [28], and ZnPP change would argue for an improvement in zinc status which plasma zinc was not able to reflect.

Inadequate intake of dietary energy, protein and micronutrients and frequent infections among children in developing countries have been suggested as causes of growth faltering [29]. In our trial, both preparations of milk provided 1890 kJ of energy and 20 g of dairy protein, and yet micronutrient fortified group showed improved weight and height gain. These findings would suggest that energy and protein without micronutrients is not sufficient to reverse the effects on growth, and providing specific micronutrients results in a better weight and height gain, and reversal of growth faltering over and above of macronutrients [30]. Growth faltering thus potentially is a functional outcome of a combination of both protein energy malnutrition and multiple micronutrients deficiency, which may explain lack of impact on growth in a recent zinc fortification trial [31]. The dietary intake data of enrolled children are presented in supplementary Tables S2, S3 and S4.

Finally, improvement in body iron stores, decrease in anemia and improvement in growth points towards a global improvement in child's health - a functional endpoint of multiple metabolic processes. This global improvement could be due to a combination of effects of individual constituents of the intervention and/or synergistic effects among the components. Given the design of the study we cannot attribute effects to a specific component but only describe the collective impact of the specific micronutrient bundle tested as the experimental intervention in this study.

In our study, we had criteria to exclude children allergic to milk; however, no such child was encountered. The results of the study cannot be generalized to populations, which may have high rates of lactose intolerance or milk allergy. These conditions in preschool children are rare in most developing country populations especially in Asia. The findings of this study cannot be directly extrapolated to fortification of other foods with similar bundle as the absorption of nutrients may vary with the kind of food being fortified.

The study provides evidence that delivery of critical micronutrients especially zinc and iron via food based vehicle, milk in this instance, is a feasible option and produces impact on growth, anemia and iron status similar to or better than that observed in supplementation trials. It provides a potential strategy for achieving Millennium Development Goals [32] targeted for reduction in mortality, morbidity and malnutrition among children. Further investigation of the effectiveness of the micronutrient-fortified milk is warranted, including cost-effectiveness before any program recommendation can be made.

## Supporting Information

Table S1 Effect of fortification on anthropometric parameters among fortified milk vs. control milk group children at mid-study (after 6 months of intervention).
(0.04 MB DOC)

Table S2 Macronutrient and micronutrient intake of the enrolled children at baseline.
(0.03 MB DOC)

Table S3 Macronutrient and micronutrient intake (including the intake from milk supplement) of the enrolled children at mid-study (after 6 months of intervention).
(0.03 MB DOC)

Table S4 Macronutrient and micronutrient intake (including the intake from milk supplement) of the enrolled children at end-study (after 12 months of intervention).
(0.03 MB DOC)

Checklist S1 Consort Statement 2001 - Checklist.
(0.06 MB DOC)

Protocol S1 Protocol for Efficacy Study of Milk Fortified with Bifidobacillus lactis HNO19 and Oligosaccharides or Zinc and Iron and other Micronutrients.
(0.27 MB DOC)
