# Supplementary material for: Micronutrient Fortified Milk Improves Iron Status, Anemia and Growth among Children 1–4 Years: A Double Masked, Randomized, Controlled Trial
Source: PLoS One. 2010 Aug 13;5(8):e12167. doi: 10.1371/journal.pone.0012167 (PMC2921413; doi:10.1371/journal.pone.0012167)
Supplement: Table S1 — Effect of fortification on anthropometric parameters among fortified milk vs. control milk group children at mid-study (after 6 months of intervention). (0.04 MB DOC) [file pone.0012167.s001.doc]

**Table S1. Effect of fortification on anthropometric parameters among fortified milk vs. control milk group children at mid-study (after 6 months of intervention)**

| **Variables** | **MN**  **(n=257)^a^** | | **Co**  **(n=257)^a^** | **Difference of means (95% CI)** | **p value** |
| --- | --- | --- | --- | --- | --- |
| **Weight velocity**^c^ (kg/yr) | 2.62±1.11^b^ | | 2.42±0.97 | 0.21 (0.03 to 0.39) | 0.02 |
| **Height velocity**^d^ (cm/yr) | 1.31±0.55 | | 1.20±0.48 | 0.11 (0.016 to 0.195) | 0.02 |
| **Change in Z scores between baseline and mid-study** | | | | | |
| Difference in WHZ score^e^ | | 0.62±0.77 | 0.57±0.73 | 0.05(-0.08 to 0.18) | 0.44 |
| Difference in WAZ score^f^ | | 0.48±0.56 | 0.36±0.52 | 0.12 (0.02 to 0.21) | 0.014 |
| Difference in HAZ score^g^ | | 0.15±0.44 | 0.02±0.38 | 0.13 (0.06 to 0.19) | <0.001 |

^a^End-study anthropometry data were available for 257 and 257 children in the MN group and in the Co group respectively;

^b^Mean ± SD;

^c^ Rate of gain in body weight over a period of 1 year of intervention;

^d^Rate of gain in height over a period of 1 year of intervention;

^e^Weight for height Z-score;

^f^Weight for age Z-score;

^g^Height for age Z-score
