# Supplementary material for: Micronutrient Fortified Milk Improves Iron Status, Anemia and Growth among Children 1–4 Years: A Double Masked, Randomized, Controlled Trial
Source: PLoS One. 2010 Aug 13;5(8):e12167. doi: 10.1371/journal.pone.0012167 (PMC2921413; doi:10.1371/journal.pone.0012167)
Supplement: Table S2 — Macronutrient and micronutrient intake of the enrolled children at baseline. (0.03 MB DOC) [file pone.0012167.s002.doc]

**Table S2.** Macronutrient and micronutrient intake of the enrolled children at baseline

| **Nutrients** | **MN**  **(n=316)** | **CO**  **(n=316)** | **p value** |
| --- | --- | --- | --- |
| Energy | 653.9±294.7 | 654±316.39 | 0.99 |
| Fat | 17.68±9.02 | 18.19±9.29 | 0.49 |
| Protein | 14.03±6.78 | 14.09±7.63 | 0.91 |
| Carbohydrate | 115.21±52.07 | 116.57±56.25 | 0.75 |
| Iron | 5.35±3.33 | 5.25±3.25 | 0.71 |
| Zinc | 2.42±1.79 | 2.43±1.75 | 0.92 |
