# Supplementary material for: Micronutrient Fortified Milk Improves Iron Status, Anemia and Growth among Children 1–4 Years: A Double Masked, Randomized, Controlled Trial
Source: PLoS One. 2010 Aug 13;5(8):e12167. doi: 10.1371/journal.pone.0012167 (PMC2921413; doi:10.1371/journal.pone.0012167)
Supplement: Table S3 — Macronutrient and micronutrient intake (including the intake from milk supplement) of the enrolled children at mid-study (after 6 months of intervention). (0.03 MB DOC) [file pone.0012167.s003.doc]

**Table S3.** Macronutrient and micronutrient intake (including the intake from milk supplement) of the enrolled children at mid-study (after 6 months of intervention)

| **Nutrients** | **MN**  **(n=316)** | **CO**  **(n=316)** | **p value** |
| --- | --- | --- | --- |
| Energy | 898.56±302.23 | 899.69±273.62 | 0.96 |
| Fat | 25.63±12.94 | 27.40±13.39 | 0.12 |
| Protein | 26.84±9.33 | 26.83±8.08 | 0.98 |
| Carbohydrate | 142.53±52.28 | 140.45±46.45 | 0.63 |
| Iron | 18.04±4.59 | 9.80±3.66 | <0.001 |
| Zinc | 12.34±3.07 | 5.78±1.76 | <0.001 |
