# Supplementary material for: Micronutrient Fortified Milk Improves Iron Status, Anemia and Growth among Children 1–4 Years: A Double Masked, Randomized, Controlled Trial
Source: PLoS One. 2010 Aug 13;5(8):e12167. doi: 10.1371/journal.pone.0012167 (PMC2921413; doi:10.1371/journal.pone.0012167)
Supplement: Table S4 — Macronutrient and micronutrient intake (including the intake from milk supplement) of the enrolled children at end-study (after 12 months of intervention). (0.03 MB DOC) [file pone.0012167.s004.doc]

**Table S4.** Macronutrient and micronutrient intake (including the intake from milk supplement) of the enrolled children at end-study (after 12 months of intervention)

| **Nutrients** | **MN**  **(n=316)** | **CO**  **(n=316)** | **p value** |
| --- | --- | --- | --- |
| Energy | 876.72±296.44 | 837.34±269.14 | 0.11 |
| Fat | 23.37±14.01 | 24.49±14.80 | 0.38 |
| Protein | 22.64±9.25 | 20.76±7.48 | 0.01 |
| Carbohydrate | 150.79±50.30 | 145.77±47.51 | 0.24 |
| Iron | 17.69±4.53 | 8.66±3.65 | <0.001 |
| Zinc | 12.23±2.92 | 5.36±1.97 | <0.001 |
